# Dermatomyositis associated with prostate adenocarcinoma with neuroendocrine differentiation

**DOI:** 10.1186/s12894-020-00779-z

**Published:** 2021-01-07

**Authors:** Hideyuki Minagawa, Taketo Kawai, Akihiko Matsumoto, Katsuhiro Makino, Yusuke Sato, Kenji Nagasaka, Masami Tokura, Nao Tanaka, Eisaku Ito, Yuta Yamada, Masaki Nakamura, Daisuke Yamada, Motofumi Suzuki, Takashi Murata, Haruki Kume

**Affiliations:** 1 Department of Urology, Ome Municipal General Hospital, 4-16-5, Higashiome Ome, Ome, Tokyo, 1980042 Japan; 2 Department of Urology, Graduate School of Medicine, The University of Tokyo, 7-3-1, Hongo, Bunkyo-ku, Tokyo, 1138655 Japan; 3 Department of Rheumatology, Ome Municipal General Hospital, 4-16-5, Higashiome Ome, Ome, Tokyo, 1980042 Japan; 4 Department of Pathology, Ome Municipal General Hospital, 4-16-5, Higashiome Ome, Ome, Tokyo, 1980042 Japan

**Keywords:** Dermatomyositis, Prostate cancer, Neuroendocrine differentiation, Dysphagia, Glucocorticoid, ADT, EP, Docetaxel, Abiraterone, Enzalutamide

## Abstract

**Background:**

Although it is known that malignancies can be associated with dermatomyositis, there are few reports on dermatomyositis associated with prostate cancer with neuroendocrine differentiation.

**Case presentation:**

A 63-year-old man visited our hospital due to pollakiuria. High levels of PSA and NSE were observed, and prostate biopsy revealed an adenocarcinoma with neuroendocrine differentiation. Multiple metastases to the lymph nodes, bones, and liver were identified, and androgen deprivation therapy (ADT) was started immediately. Following 2 weeks of treatment, erythema on the skin, and muscle weakness with severe dysphagia appeared. The patient was diagnosed with dermatomyositis, and high-dose glucocorticoid therapy was initiated. ADT and subsequent chemotherapy with etoposide and cisplatin (EP) were performed for prostate cancer, which resulted in decreased PSA and NSE and reduction of all metastases. After the initiation of EP therapy, dermatomyositis improved, and the patient regained oral intake function. Although EP therapy was replaced by docetaxel, abiraterone, and enzalutamide because of adverse events, no cancer progression was consistently observed. Dermatomyositis worsened temporarily during the administration of abiraterone, but it improved upon switching from abiraterone to enzalutamide and dose escalation of glucocorticoid.

**Conclusions:**

We successfully treated a rare case of dermatomyositis associated with prostate adenocarcinoma with neuroendocrine differentiation.

## Background

Approximately 20% of all dermatomyositis cases are accompanied by malignancies [1–3]. Furthermore, among patients with dermatomyositis, those with malignancies have a poor prognosis [1, 2]. Although there are several reports of prostate cancer with dermatomyositis [1, 3, 4], we are aware of only one case of neuroendocrine prostate cancer with dermatomyositis [5], who responded poorly to treatment and died within 4 months after initiation of treatment. Here, we report a case of long-term survival of dermatomyositis with severe dysphagia which was associated with prostate adenocarcinoma with neuroendocrine differentiation.

## Case presentation

**Fig. 1:**
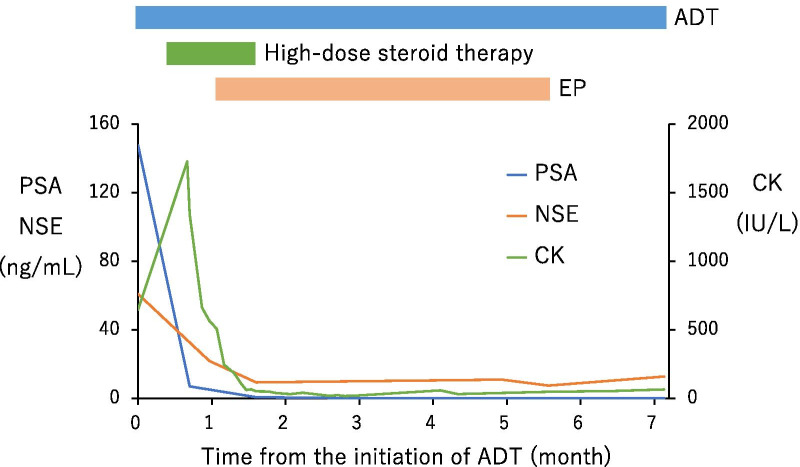
Temporal changes in PSA, NSE and CK. The treatments performed are indicated above the graph

**Fig. 2:**
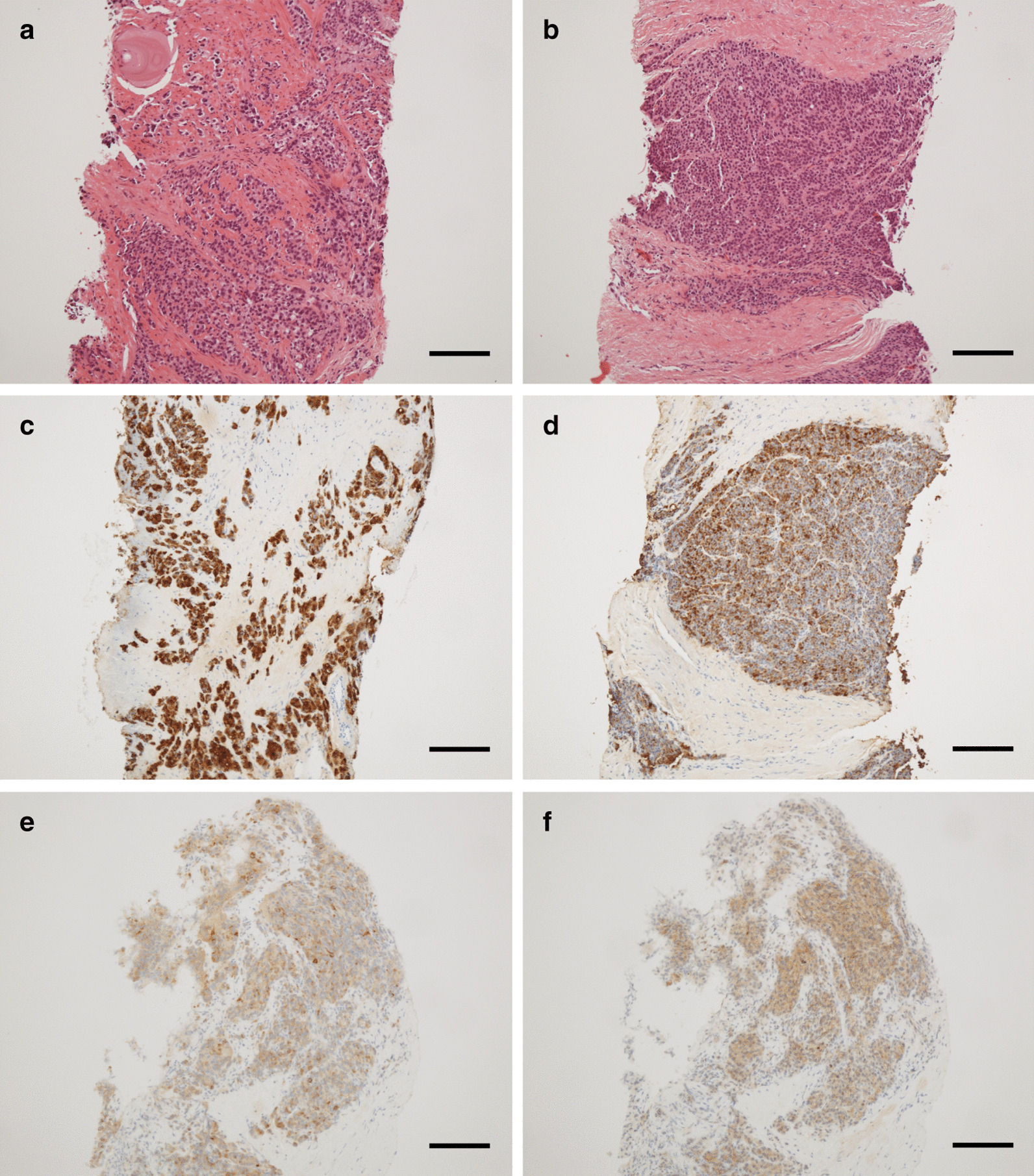
Immunohistochemical findings of prostate biopsy; hematoxylin and eosin (**a**, **b**), PSA (**c**, **d**), synaptophysin (**e**), and NCAM1/CD56 (**f**). Hematoxylin and eosin staining reveals adenocarcinoma with Gleason score 4 + 5 (**a**) and components of neuroendocrine differentiation (**b**). The components of typical adenocarcinoma (**c**) and neuroendocrine differentiation (**d**) are highly and weakly positive for PSA, respectively. The components of neuroendocrine differentiation are positive for synaptophysin (**e**) and NCAM1/CD56 (**f**). The bar indicates 100 μm

**Fig. 3:**
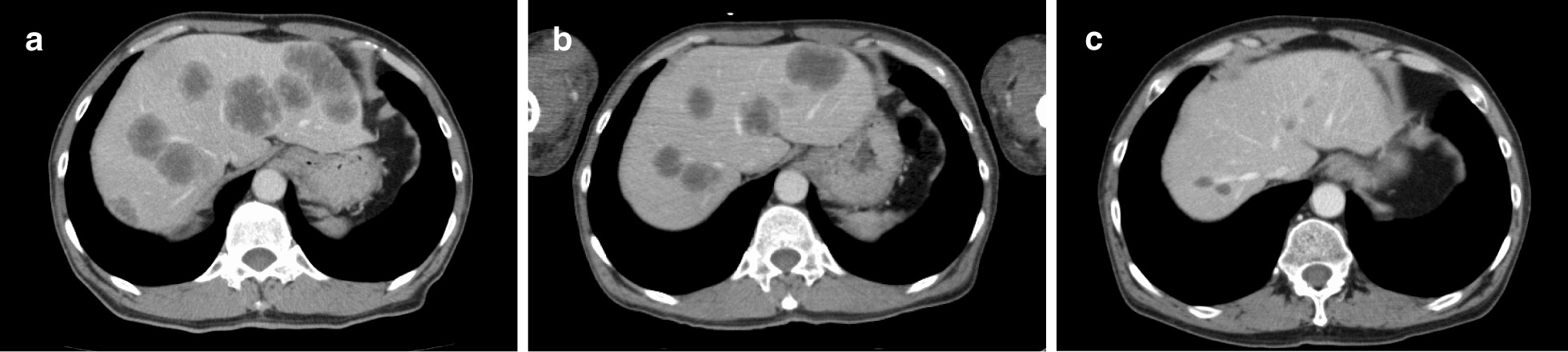
CT findings of liver metastases before ADT (**a**), before EP therapy (**b**), and after 4 courses of EP therapy (**c**)

A 63-year-old man visited our hospital complaining of frequent urination and hesitancy, which had worsened a year prior to the visit. He also complained of muscle weakness and pain in the upper arms. Blood tests revealed abnormally high PSA (147.7 ng/mL), NSE (60.9 ng/mL), and CK (647 IU/L) (Fig. 1). Prostate biopsy revealed adenocarcinoma with Gleason score 4 + 5 accompanied by neuroendocrine differentiation, which was positive for PSA, synaptophysin, and NCAM1/CD56 by immunohistochemistry (Fig. 2). Contrast-enhanced computed tomography (CT) revealed prostate cancer which showed invasion to the bladder, seminal vesicles, and rectum. Multiple metastases to the liver (Fig. 3a), pelvic lymph nodes, and bone were observed. The patient was diagnosed with prostate adenocarcinoma with neuroendocrine differentiation, cT4N1M1. At the time of diagnosis, the use of abiraterone and docetaxel for high-risk metastatic hormone-sensitive prostate cancer (HSPC) was not yet approved in medical insurance in Japan. Therefore, combined androgen blockade (CAB) therapy with surgical castration and bicalutamide was initiated as the most intense treatment available in Japan at that time for high-risk metastatic HSPC. Chemotherapy with etoposide and cisplatin (EP) was not performed at this time because pure adenocarcinoma components dominated, few with neuroendocrine differentiation in the prostate biopsy.

**Fig. 4:**
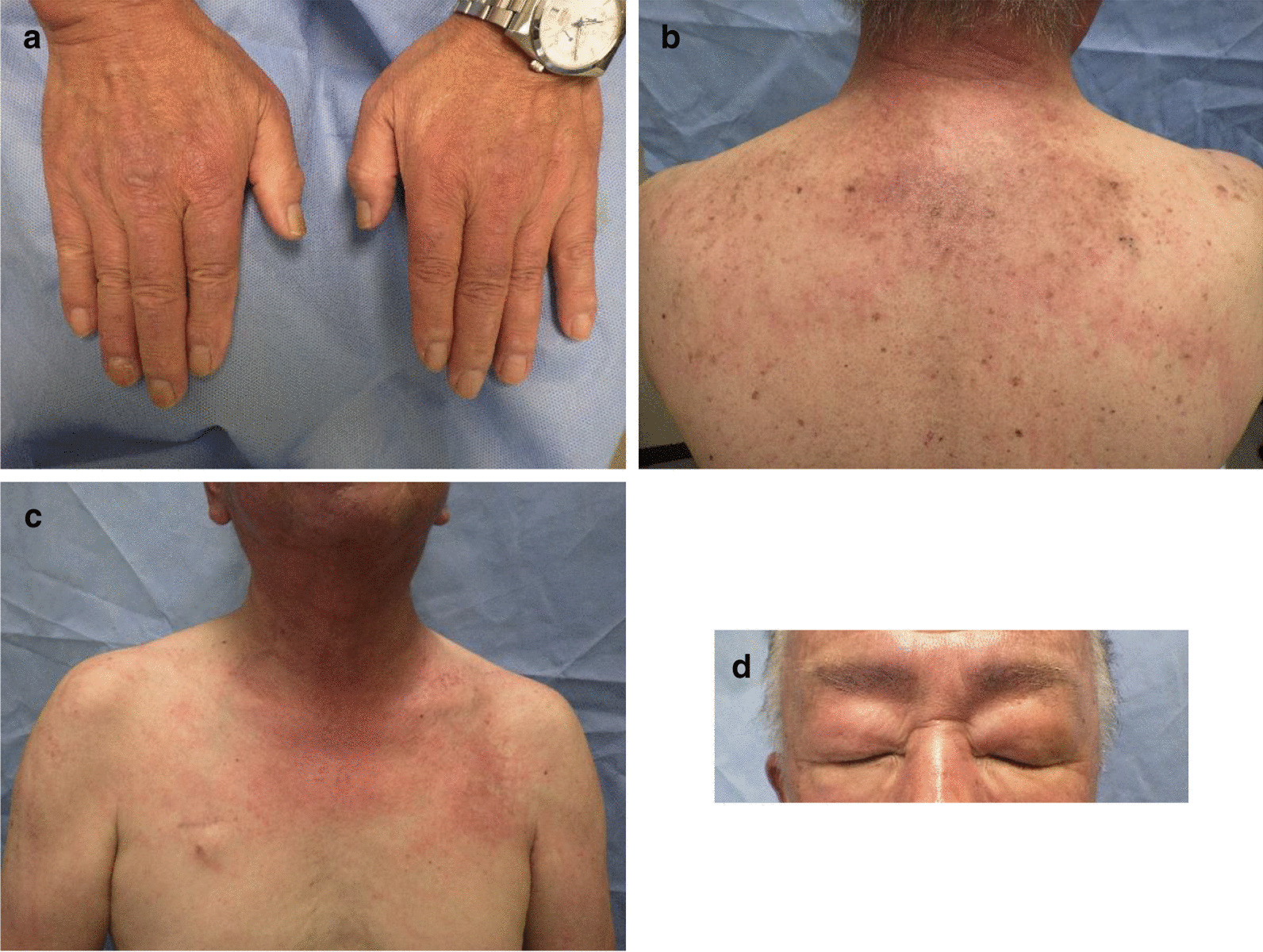
Various skin symptoms observed in the patient. Erythema on the back of the finger joint (Gottron’s sign) (**a**), erythema from shoulder to upper back (shawl sign) (**b**), erythema around the neck (V-neck sign) (**c**), and edematous erythema on the bilateral eyelids (heliotrope rash) (**d**)

Two weeks later, dysphagia, edema of both upper limbs, and erythema on the skin were identified. Bicalutamide was discontinued and high-dose glucocorticoid therapy with methylprednisolone (mPSL) was initiated at a dose of 125 mg/day intravenously (IV) for the first 3 days, 80 mg/day IV for the next 3 days, and 40 mg/day IV for the next 3 days. At this time, the symptoms were suspected to be bicalutamide-induced adverse events. One week after the initiation of mPSL, dysphagia and erythema of the skin and muscle weakness of both arms did not improve. Physical examination revealed erythema on the back of the finger joints (Gottron’s sign) (Fig. 4a), from the shoulder to the upper back (shawl sign) (Fig. 4b) and around the neck (V-neck sign) (Fig. 4c), and edematous erythema on the bilateral eyelids (heliotrope rash) (Fig. 4d). Blood tests revealed abnormally high values of CK (1727 IU/L, Fig. 1), aldolase (14.5 U/L), and myoglobin (435.8 ng/mL). Anti-nuclear antibody was positive at titers of 1:640, anti-ARS antibody was negative, and anti-TIF1-γ antibody was positive. Based on these findings, the patient was diagnosed with malignancy-associated dermatomyositis. He was presented with dysphagia, which was severe and required temporary fasting and central parenteral nutrition. Glucocorticoid administration was changed from mPSL to prednisolone (PSL) at a dose of 60 mg/day IV.

Four weeks after the start of androgen deprivation therapy (ADT), CT showed residual multiple liver metastases (Fig. 3b), although PSA and NSE decreased to 7.0 ng/mL and 21.9 ng/mL, respectively (Fig. 1). Then, EP therapy (etoposide 100 mg/m^2^ and cisplatin 20 mg/m^2^, every 3 weeks) was commenced, resulting in a rapid decrease of PSA and NSE to < 1 ng/mL and < 13 ng/mL, respectively (Fig. 1). Two weeks after EP therapy started, swallowing function improved with a decrease in CK (Fig. 1) and oral intake became possible. PSL administration was gradually tapered to 5 mg/day.

After 4 courses of EP therapy, CT showed marked reduction of the primary tumor and all metastases including those in the liver (Fig. 3c), but EP therapy was discontinued due to grade 3 malaise. Although the metastases had shrunk, they did not disappear, and we supposed that a small amount of cancer progression could lead to a relapse of dermatomyositis. The patient was therefore switched to docetaxel therapy (70 mg/m^2^, every 3 weeks). After 4 courses, docetaxel was also discontinued due to grade 3 malaise, and abiraterone acetate treatment was initiated. Abiraterone acetate treatment was ceased after one month because erythema on his face and extremities and serum CK level worsened (246 IU/L). Enzalutamide treatment and dose escalation of PSL to 30 mg/day were started, and dermatomyositis improved. PSA and NSE were consistently low, and no progressions were observed during the administration of docetaxel, abiraterone, and enzalutamide. At present, 31 months after the start of enzalutamide administration, no cancer progression has been observed. Dermatomyositis is in remission, and the patient is on PSL at 10 mg/day.

## Discussion and conclusions

It is difficult to obtain a good therapeutic effect in patients with dermatomyositis complicated by malignant tumors, as long as the tumor is present; conversely, radical treatment of malignancy may improve muscle and skin symptoms. Therefore, treatment of malignancy is prioritized in order to improve dermatomyositis [3]. In the present case, dermatomyositis was improved by treatment for prostate cancer in addition to high-dose glucocorticoid therapy. ADT was initially performed to treat prostate cancer with neuroendocrine differentiation and the treatment had limited effects on dermatomyositis, and dysphagia became apparent. Although dermatomyositis tends to improve with high-dose glucocorticoid therapy, oral intake was possible after EP therapy was commenced. These findings suggest that EP therapy was required to address the component of neuroendocrine differentiation.

Pure neuroendocrine prostate cancer (including small cell carcinoma and large cell carcinoma) has a poor prognosis. However, it is controversial whether neuroendocrine differentiation in adenocarcinomas worsens the prognosis [6, 7]. In the present case, multiple liver metastases were observed from the first visit, suggesting that the cancer was aggressive. ADT was followed by EP therapy and other strong treatments with docetaxel, abiraterone, and enzalutamide before the onset of castration resistance. We believe that the ability to control aggressive cancers with these treatments may have led to an improvement in disease activity of dermatomyositis.

In conclusion, we encountered a rare case of dermatomyositis associated with prostate adenocarcinoma with neuroendocrine differentiation. The patient was successfully treated with ADT and subsequent EP therapy for prostate cancer and high-dose glucocorticoid therapy for dermatomyositis.

## Data Availability

All data generated or analyzed during this study are included in this published article and available from the corresponding author on reasonable request.

## Abbreviations

PSA: Prostate specific antigen
NSE: Neuron specific enolase
CK: Creatinine kinase
NCAM1: Neural cell adhesion molecule 1
